# Use of Carboxyhemoglobin as an Early Sign of Oxygenator Dysfunction in Patients Supported by Extracorporeal Membrane Oxygenation

**DOI:** 10.3389/fmed.2022.893642

**Published:** 2022-04-29

**Authors:** Vladimir L. Cousin, Raphaël Giraud, Benjamin Assouline, Ivo Neto Silva, Karim Bendjelid

**Affiliations:** ^1^Intensive Care Unit, Geneva University Hospital, Geneva, Switzerland; ^2^Geneva Hemodynamic Research Group, Geneva University, Geneva, Switzerland

**Keywords:** carboxyhemoglobin, ECMO, critical care, hemolysis, oxygenator clotting

## Abstract

**Introduction:**

Plasma free hemoglobin is the gold standard for monitoring hemolysis in extracorporeal membrane oxygenation (ECMO) but its routine use has some limitations. Carboxyhemoglobin (HbCO) is also a marker of intravascular hemolysis. We aimed to investigate HbCO as a marker of both hemolysis and oxygenator dysfunction in patients supported by ECMO.

**Methods:**

Retrospective analysis of patients on ECMO in an adult ICU in a tertiary hospital. HbCO was recorded every 6 h in the 48 h before and after oxygenator change in adult patients on ECMO support with an oxygenator dysfunction and replacement.

**Results:**

The investigation of 27 oxygenators replacements in 19 patients demonstrated that HbCO values progressively increased over time and then significantly decreased after oxygenator change. Median oxygenator lifespan was 14 days [interquartile range (IQR) 8–21] and there was no correlation between HbCO and oxygenator lifespan [Spearman coefficient 0.23 (*p* = 0.23)]. HbCO values at oxygenator change [HbCO median 2.7 (IQR 2.5–3.5)] were significantly higher than the HbCO values 1 week before [HbCO median 2.07 (IQR 1.86–2.8)] (*p* value < 0.001).

**Conclusion:**

Our data highlight the potential role of HbCO as a novel marker for ECMO oxygenator dysfunction.

## Introduction

Extracorporeal membrane oxygenation (ECMO) support is associated with a risk of hemolysis (1). In the present setting, the hemolysis phenomenon could be a sign of oxygenator dysfunction associated with continuous disseminated intravascular coagulation (DIC). To detect and monitor hemolysis, plasma-free hemoglobin monitoring is recommended by the Extracorporeal Life Support Organization (2). However, the availability of free hemoglobin monitoring has limitations as it is not universally available, and the result may take several hours to be obtained. Under such conditions, carboxyhemoglobin (HbCO) levels seem to be an accessible and reliable marker of hemolysis (3, 4). During hemolysis, heme is degraded by heme oxygenase into biliverdin, free iron and carbon monoxide (5). Then, carbon monoxide binds with hemoglobin to form HbCO. There are several case reports of elevated HbCO in cases of hemolysis and oxygenator dysfunction in the current literature (6–8). However, there are currently no studies looking at the use of HbCO values as an early additional marker of ECMO oxygenator dysfunction.

Therefore, the aim of our study was to evaluate the evolution of HbCO values over time before and after oxygenator replacement. HbCO monitoring could be used as a marker for oxygenator dysfunction in patients on ECMO as surrogate for free Hb, especially in hospitals that do not have a readily access to free Hb.

## Methods

The present study was conducted during a 20-month period (January 2020–August 2021) in the intensive care unit at Geneva University Hospital. The local ethics committee approved the study and waived the informed consent (BASEC number: 2020-00917). Patients with refractory cardiogenic shock supported by veno-arterial (VA)-ECMO or with refractory hypoxemia supported by veno-venous (VV)-ECMO were screened. HbCO measurement is currently performed in all patients in the unit by the blood gas analyzer.

The exclusion criteria were patients without HbCO available at least one week before ECMO implantation, patients with missing data on oxygenator dysfunction and the inability to collect HbCO before and/or after oxygenator change. ECMO use was based on ELSO recommendations for both VA and VV support (2). In the case of SARS-CoV 2 infection, our center follows an institutional algorithm validated by the Swiss Society of Intensive Care Medicine (9). ECMO management is summarized in the Supplementary Material. Oxygenator were replaced in cases low post-oxygenator PaO_2_, increase of transmembrane pressure, visible oxygenator clot with diminished ECMO flow, consumptive coagulopathy or hemolysis. Such change was discussed between ECMO team members and carried out in the unit (2, 10).

Carboxyhemoglobin was recorded every 6 h in the 48 h before and after oxygenator change. Time at oxygenator change is labeled H0, as the first blood gazes in the hour after the oxygenator change. HbCO was measured using a blood gas analyzer [Radiometer ABL800 FLEX, Radiometer ABL90 FLEX PLUS or ABL90 FLEX (Radiometer RSCH GmbH, Thalwil, CH)]. We also recorded hemoglobin (Hb, g/L), lactate (mmol/L), pH, PaO_2_ (kPa), and PaCO_2_ (kPa) at each time point. We determined a baseline HbCO using the mean HbCO value seven days before the oxygenator change (range per patient 3–12), used as an internal control for baseline HbCO.

Continuous variables were described using medians with interquartile ranges (IQRs), and binomial variables were described using proportions. Values of HbCO were compared using the Wilcoxon signed-rank test between different time points. Spearman correlation was performed between oxygenator lifespan and HbCO. A *p* value ≤0.05 was considered significant. Statistical analyses were carried out using Stata Software (StataCorp, College Station, TX, United States).

## Results

**FIGURE 1:**
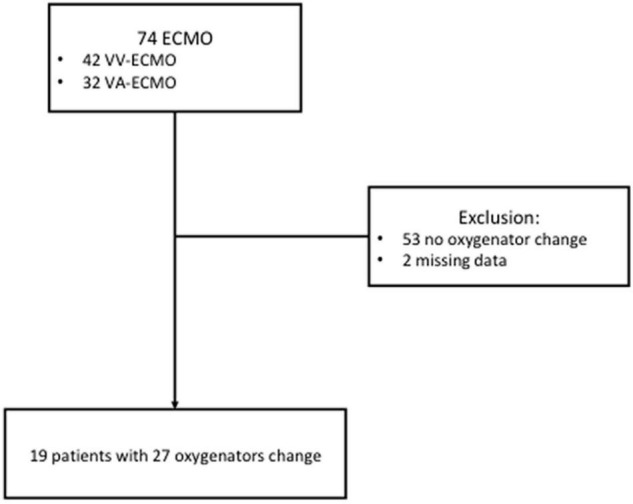
Study flow chart.

**FIGURE 2:**
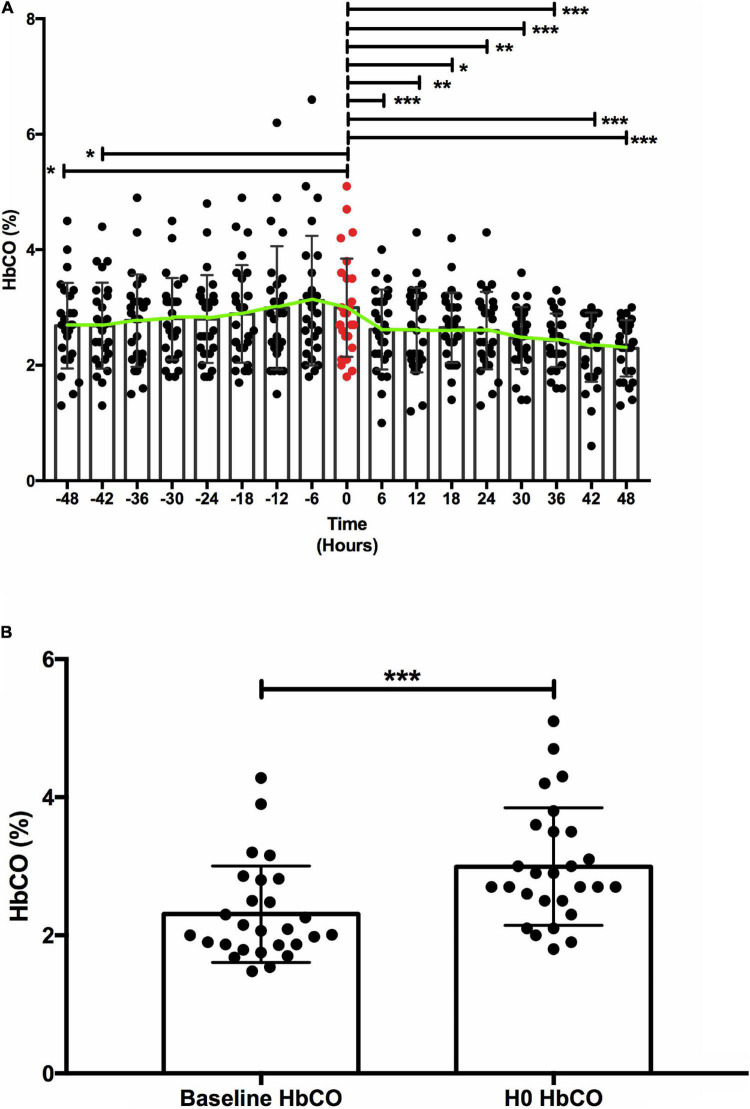
Carboxyhemoglobin (HbCO) evolution. (A) HbCO evolution before/after oxygenator replacement. (B) Carboxyhemoblobin baseline compared to carboxyhemoglobin at oxygenator replacement. (A) Comparison of mean (±SD) HbCO before and after oxygenator replacement. Green line connects HbCO mean at each time point. The red value represents the HbCO value at the oxygenator change, at H0. Y axis: HbCO (%), X axis: time in hours. * < 0.05 ** < 0.01 *** < 0.001. (B) H0 HbCO was significantly higher [median 2.7 (IQR 2.5–3.5)] than baseline HbCO [median 2.07 (IQR 1.86–2.8)], with a *p* value < 0.001. Y axis: HbCO (%), X axis: time-point *** < 0.001.

During the study period, all patients receiving ECMO support were screened (Figure 1). The final population included 19 patients with 27 oxygenator replacements (range per patient 1–5). Patient characteristics are summarized in Table 1. Figure 2A shows the evolution of HbCO in the 48 h before and after the oxygenator change. Noticeably, after oxygenator change, all HbCO values were significantly lower than H0 HbCO. When compared to baseline HbCO, the H0 HbCO level was significantly higher, as shown in Figure 2B. Median oxygenator lifespan was 14 days (IQR 8–21). We did not find a correlation between HbCO and oxygenator lifespan, with a Spearman coefficient of 0.23 (*p* = 0.23). When compared, the lifespans of VV- and VA-oxygenators were not significantly different: 16 days (IQR 9–21.5) for VV ECMO and 9 (IQR 4–11) for VA ECMO (*p* = 0.08). HbCO evolution between both groups (Supplementary Figure 1) shows that HbCO was higher in the case of VV ECMO.

**TABLE 1:** Population characteristics.

Age (years)	59 (IQR 55–65)
Sex (M)	13 (68%)
Diagnosis at time of ECMO cannulation	15 Covid-19 ARDS 3 Cardiogenic Shock (ischemic) 1 Massive Hemoptysis
ECMO support	16 Veno-venous (VV)
	3 Veno-arterial (VA)
Length of stay in ICU (days)	38 (IQR 26–69)
Length of ECMO support (days)	28 (IQR 24–43)
Outcome (deceased)	11 (57%)
Oxygenator lifespan (days)	14 (IQR 8-21)
At oxygenator change	
HbCO (%)	2.7 (IQR 2.5–3.5)
Lactate (mmol/L)	1.1 (IQR 0.8–1.3)
Hemoglobin (g/L)	85 (IQR 77–91)
pH	7.45 (IQR 7.41–7.48)
paO2 (kPa)	8.9 (IQR 8.2–9.9)
paCO2 (kPa)	5.1 (IQR 4.5–5.4)

*ARDS, acute respiratory distress syndrome; ICU, intensive care unit; ECMO, extracorporeal membrane oxygenation; HbCO, carboxyhemoglobin.*

## Discussion

In the present study, we showed in a representative group of patients a progressive increase and subsequent decrease in HbCO around the time of oxygenator replacement, suggesting a role for HbCO values as an early marker of oxygenator-induced hemolysis.

In the case of oxygenator dysfunction, microthrombi inside the ECMO membrane could produce hemolysis and then increase HbCO levels. HbCO monitoring could then be used in situation when plasma hemoglobin is inaccurate (e.g., significant hyperbilirubinemia or hyperlipidemia, extreme hemolysis with high plasma hemoglobin levels) or unavailable. In this regard, replacing the oxygenator restored HbCO levels. Such findings are similar to the report by Hoffman et al. (7), who described the evolution of HbCO before and after oxygenator replacement, and Kimura et al., who showed an HbCO increase on ECMO and a significant reduction after ECMO removal (11). Such an increased level of HbCO might be an indicator of impending oxygenator dysfunction, able to both induce thromboembolic events and increase mortality (6–8). Importantly, oxygenator lifespan was not associated with HbCO, suggesting that a functional oxygenator will not raise HbCO. Our data suggest a role for HbCO as a warning sign in cases of oxygenation difficulties or ECMO dysfunction. A progressive increase of HbCO level could be used as a warning sign of oxygenator dysfunction. Moreover, HbCO might also be used for the diagnosis of hemolysis with other associated markers, such as bilirubin or free hemoglobin.

The present study has some limitations. First, the design was retrospective with a limited number of patients. Second, it is important to note that other factors, such as systemic inflammation, cavitation or high RPM may impact HbCO (5). Due to laboratory limitation, we could not have made correlation between HbCO and other hemolysis marker such as free hemoglobin or haptoglobin. Finally, our design was based on the comparison of the trend of HbCO in the same patient with oxygenator dysfunction (own control), without comparing HbCO in patients with and with no oxygenator change.

In conclusion, the present study highlights the role of HbCO as a novel marker and monitoring of ECMO oxygenator dysfunction. Further studies are needed to investigate the use of HbCO in clinical settings and compare HbCO levels in patients with and without oxygenator change.

## Data Availability Statement

The raw data supporting the conclusions of this article will be made available by the authors, without undue reservation.

## Ethics Statement

The studies involving human participants were reviewed and approved by BASEC number: 2020-00917. Written informed consent for participation was not required for this study in accordance with the national legislation and the institutional requirements.

## Author Contributions

VC, RG, and KB: concept and design. VC and RG: data collection. VC and KB: data analysis and draft manuscript. VC, RG, BA, IS, and KB: critically revised and approved the manuscript. All authors contributed to the article and approved the submitted version.

## Conflict of Interest

The authors declare that the research was conducted in the absence of any commercial or financial relationships that could be construed as a potential conflict of interest.

## Publisher’s Note

All claims expressed in this article are solely those of the authors and do not necessarily represent those of their affiliated organizations, or those of the publisher, the editors and the reviewers. Any product that may be evaluated in this article, or claim that may be made by its manufacturer, is not guaranteed or endorsed by the publisher.
