# Erratum: Use of carboxyhemoglobin as an early sign of oxygenator dysfunction in patients supported by extracorporeal membrane oxygenation

**DOI:** 10.3389/fmed.2023.1186498

**Published:** 2023-03-24

**Authors:** Frontiers Production Office

**Affiliations:** Frontiers Media SA, Lausanne, Switzerland

**Keywords:** carboxyhemoglobin, ECMO, critical care, hemolysis, oxygenator clotting

An omission to the funding section of the original article was made in error. The following sentence has been added: “Open access funding was provided by the University of Geneva.”

The original article has been updated.

