# Supplementary material for: Use of Carboxyhemoglobin as an Early Sign of Oxygenator Dysfunction in Patients Supported by Extracorporeal Membrane Oxygenation
Source: Front Med (Lausanne). 2022 Apr 29;9:893642. doi: 10.3389/fmed.2022.893642 (PMC9106404; doi:10.3389/fmed.2022.893642)
Supplement: Supplementary file 1 [file Data_Sheet_1.PDF]

Supplementary Material

ECMO Management

As we mentioned, ECMO cannulation was based on ESLO recommendations for both VA and VV support, and a special protocol was developed in the context of the SARS-CoV2 crisis. The final decision to set up ECMO was made by the on-call ECMO specialist in our hospital. All patients were cannulated percutaneously with echo-guided punctures. We used femoral-femoral cannulas for VA-ECMO and femoral-jugular cannulas for VV-ECMO. In the case of VA-ECMO, a catheter was systematically inserted into the superficial femoral artery to prevent lower limb ischaemia. Pump speed was adjusted to obtain adequate blood flow and adequate saturation. We used unfractionated heparin in our unit, titrated to maintain an anti-XA 0,2-0,3 for VV-ECMO and 0,3-0,4 for VA-ECMO. Experienced perfusionists checked the circuit and oxygenator daily. In the case of VA-ECMO, the use of an intra-aortic balloon pump or percutaneously inserted ventricular assist device was decided case by case by the ECMO team along with the cardiologist. Oxygenator were replaced in cases low post-oxygenator PaO_2, increase of transmembrane pressure, visible oxygenator clot with diminished ECMO flow, consumptive coagulopathy or hemolysis. Such change was discussed between ECMO team members and carried out in the unit. Such change was discussed between ECMO team members and carried out in the unit.

Supplementary Figure 1

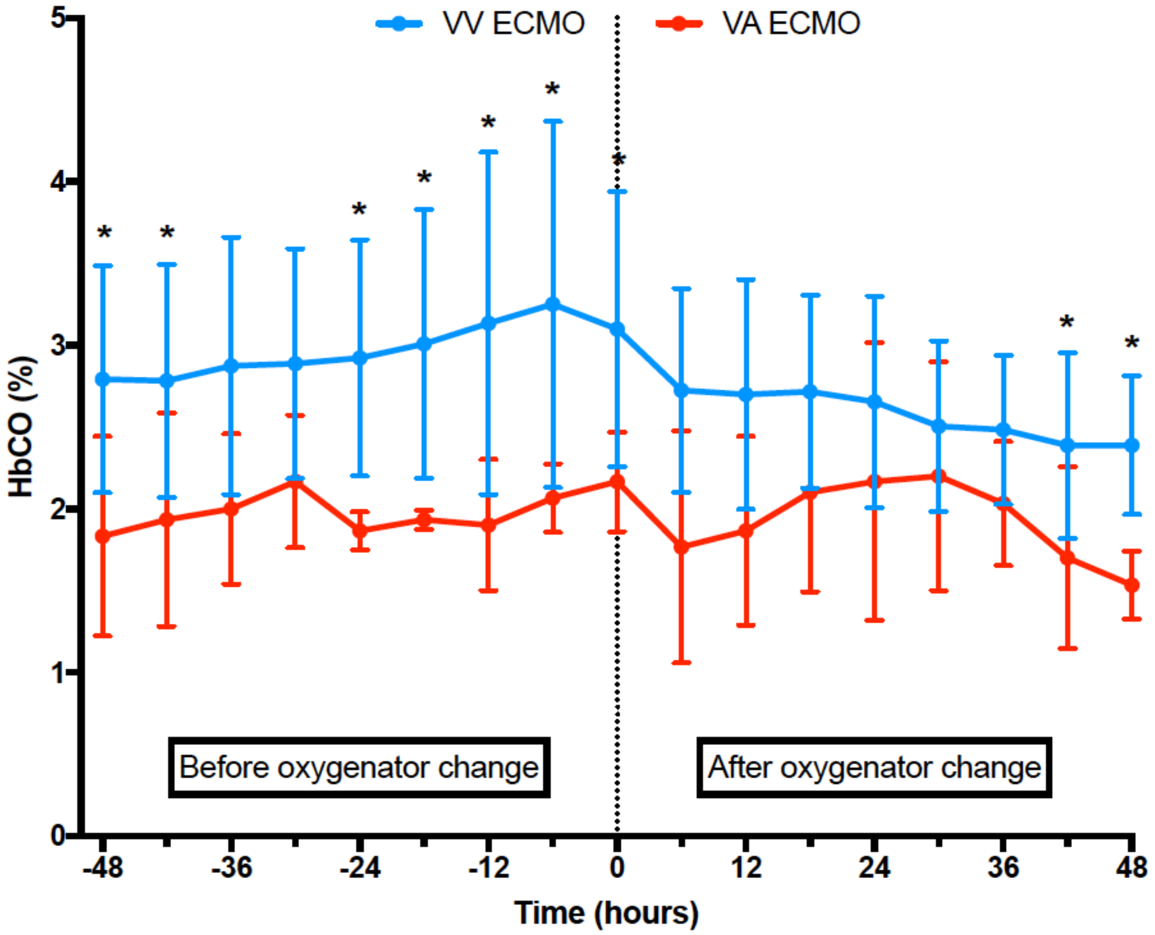

Supplementary Figure 2 Carboxyhemoglobin evolution compared between veno-venous and veno-arterial ECMO before and after oxygenator replacement

Comparison of mean (+- SD) HbCO before and after oxygenator replacement, depending on the type of ECMO support. Veno-Venous V-ECMO in blue. Veno-arterial ECMO in red. Y axis: HbCO (%), X axis: time in hours. *<0,05
